# Definition of the Anti-inflammatory Oligosaccharides Derived From the Galactosaminogalactan (GAG) From *Aspergillus fumigatus*

**DOI:** 10.3389/fcimb.2019.00365

**Published:** 2019-11-06

**Authors:** Markus Gressler, Christoph Heddergott, Inés C. N'Go, Giorgia Renga, Vasilis Oikonomou, Silvia Moretti, Bernadette Coddeville, Joana Gaifem, Ricardo Silvestre, Luigina Romani, Jean-Paul Latgé, Thierry Fontaine

**Affiliations:** ^1^Unité des Aspergillus, Institut Pasteur, Paris, France; ^2^Department of Experimental Medicine, Università degli Studi di Perugia, Perugia, Italy; ^3^Unité de Glycobiologie Structurale et Fonctionnelle (UGSF) UMR 8576 CNRS, Université de Lille, Lille, France; ^4^Life and Health Sciences Research Institute (ICVS), School of Medicine, University of Minho, Braga, Portugal; ^5^ICVS/3B's-PT Government Associate Laboratory, Braga, Portugal

**Keywords:** galactosaminogalactan, *Aspergillus fumigatus*, IL-Ra, anti-inflammatory response, glycodrug

## Abstract

Galactosaminogalactan (GAG) is an insoluble aminosugar polymer produced by *Aspergillus fumigatus* and has anti-inflammatory properties. Here, the minimum glycosidic sequences required for the induction of IL-1Ra by peripheral blood mononuclear cells (PBMCs) was investigated. Using chemical degradation of native GAG to isolate soluble oligomers, we have found that the de-*N*-acetylation of galactosamine residues and the size of oligomer are critical for the *in vitro* immune response. A minimal oligomer size of 20 galactosamine residues is required for the anti-inflammatory response but the presence of galactose residues is not necessary. In a Dextran sulfate induced colitis mouse model, a fraction of de-*N*-acetylated oligomers of 13 < dp < 20 rescue inflammatory damage like the native GAG polymer in an IL-1Ra dependent pathway. Our results demonstrate the therapeutic suitability of water-soluble GAG oligosaccharides in IL-1 mediated hyper-inflammatory diseases and suggest that α-1,4-galactosamine oligomers chemically synthesized could represent new anti-inflammatory glycodrugs.

## Introduction

The galactosaminogalactan (GAG) is a polysaccharide produced by the human fungal pathogen, *Aspergillus fumigatus* which is a major virulence factor for this fungal species. GAG mediates the adherence of mycelium to abiotic or host-cell surfaces within biofilm and protects the fungus against host-cell damages (Gravelat et al., 2013; Beaussart et al., 2015; Lee et al., 2015). In mouse model of aspergillosis, *intra-nasally* administration of GAG promotes fungal infection and directly affects the innate immune response resulting in an inhibition of the protective T-helper (Th) 1 and regulatory T cells (Treg) as well as a promotion of Th2 responses (Fontaine et al., 2011). In addition, GAG induces apoptosis in neutrophils (Robinet et al., 2014). GAG did not induce any of the proinflammatory cytokines TNFα, IL-6, IL-8, IFNγ, IL-17, and IL-9 neither the anti-inflammatory cytokine IL-10 (Gresnigt et al., 2014). However, GAG reduced the production of Th cytokines IL-17, IL-22, and IFNγ in *Aspergillus* stimulated PBMCs. Most importantly, GAG inhibits the IL-1 bioactivity by the induction of the production of high amounts of IL-1 receptor antagonist (IL-1Ra) (Gresnigt et al., 2014). The capacity of GAG to induce IL-1Ra is responsible for its potent anti-inflammatory properties favoring *Aspergillus* infections (Gresnigt et al., 2014). The anti-inflammatory potential of GAG has been also shown in experimental dextran sodium sulfate (DSS)-induced colitis in mice with chronic granulomatous disease (CGD). The intraperitoneal administration of GAG resulted in the improvement of clinical signs of colitis and of inflammatory lesions in both wild-type and CGD mice. The effects of GAG on CGD colitis were similar to those of IL-1Ra administration (Gresnigt et al., 2014), making it a potent alternative to tackle chronic inflammatory diseases.

GAG is a linear heterogeneous high molecular weight polymer with an average size of 100 kDa and it is composed of α-1,4-linked galactopyranose (Gal*p*), α-1,4-linked galactosamine (GalNH_2_), and α-1,4-linked N-acetylgalactosamine (GalNAc) residues. Monosaccharides are randomly distributed along the chain (Fontaine et al., 2011; Lee et al., 2016). Due to the heterogeneity of the molecule and the absence of clear repeating unit, the oligosaccharides responsible for the immunological functions of GAG are unknown. It has been only shown that the deacetylation of the GAG is essential for the adherence and the virulence of the fungus (Lee et al., 2016). However, the degree of acetylation and the size of the oligosaccharide responsible for the anti-inflammatory properties of GAG were unknown to date. For this purpose, we have isolated water-soluble oligomers that are inducers of IL-1Ra and shown that long water-soluble de-*N*-acetylated galactosamine oligomers of 13 < DP < 20 rescue inflammatory damage similarly as the GAG polymer in an IL-1Ra dependent pathway.

## Materials and Methods

### Cultivation and GAG Purification

*A. fumigatus* strain Dal (CBS144-89) was cultivated on malt-agar tubes for 3 days at 37°C. Conidia were collected with 4 ml 0.05% tween20 and vortexing of the tube. The conidia suspension was stored up to 3 weeks at 4°C.

GAG isolation and purification was carried out as previously described (Fontaine et al., 2011). Briefly, 100 ml Brian medium was inoculated with a final concentration of 5 × 10^5^ conidia per ml in 150 ml Erlenmeyer flasks. After cultivation for 3 days at 37°C and 170 rpm, the pooled supernatant of 20 cultures was collected by filtration and was adjusted to pH 3 by addition of 100 μl 12 M HCl per 100 ml supernatant. Two volumes of precooled ethanol (4°C) were added and GAG was precipitated for 3 h at 4°C. The precipitate was collected by centrifugation for 20 min at 5,000 g at 4°C and subsequently washed twice with 1/10 of the culture volume of 100 mM NaCl for 1 h under agitation (100 rpm). GAG was dialyzed against tap water and twice against purified water (24 h each) and finally lyophilized to dryness and stored at ambient temperature.

### Chemical Modification of Polysaccharides

#### De-*N*-Acetylation of Polysaccharides

GAG was suspended in 3 ml of 10 mM HCl at a final concentration of 3.33 mg/ml by sonication in plastic tubes. The de-*N*-acetylation was started by addition of 3.4 ml 18.8 M NaOH and the mixture was incubated at 100°C for up to 4–5 h. The tubes were vortexed every hour. The reaction was stopped on ice and neutralization with 12 M HCl. The mixture was buffered by 20 mM Tris, pH 7. De-*N*-acetylated GAGs (dGAG) were dialyzed against tap water and twice against dH_2_0 (24 h each) and finally lyophilized to dryness and stored at ambient temperature.

#### Acetylation of Oligosaccharides

The acetylation procedure described by Lavertu et al. (2012) was modified as following: 0.5 mg dGAG oligosaccharides (F-I and F-III) were lyophilized to dryness and were solved in 25 μl of 400 mM acetic acid and 100 μl CH_3_OH. The mixture was preincubated for 1 h at ambient temperature under agitation at 300 rpm. Acetylation was initiated by addition 3 μl acetic anhydride. After 1 h of incubation, solvents were evaporated in a desiccator overnight. The samples were desalted by repeated dissolution with 500 μl water (4 times) followed by evaporation to dryness. Samples were solved in 500 μl water and finally stored at −20°C.

#### Production of GAG Oligosaccharides

De-*N*-acetylated GAG (dGAG) was hydrolyzed by 2 M HCl at 100°C for 1.5, 3, and 4.5 h. Preliminary assays showed that 1.5 and 3 h were optimal for generating oligosaccharides of different sizes (DP 3–26). HCl was immediately evaporated after hydrolysis by drying the oligosaccharide mixture overnight under vacuum in presence of NaOH pellets. Soluble oligosaccharides were dissolved in 1 ml of 150 mM ammonium acetate, pH 4.5 and unsolved particles were removed by centrifugation (3 min, 17,000 g). The oligosaccharides were subjected to size exclusion chromatography (SEC) on a Superdex 30 column (GE Healthcare, 10 × 750 mm) equilibrated with 150 mM ammonium acetate, pH 4.5 as described (Hamer et al., 2015). The oligosaccharides were separated at a flow rate of 0.5 ml/min and detected by the Gilson 132 RI detector. The osamine amount of each fraction was determined by 3-methyl-2-benzothiazolinon hydrazone hydrochloride hydrate assay (MBTH, see below). In total eight 8 ml-fractions were obtained by pooling four 2 ml-fractions per gel permeation assays and were named as F-I to F-VIII (Figure S1). The fractions were desalted by lyophilization for 2–3 days, addition of 1 ml water and subsequent second lyophilization overnight in a Savant speed vac concentrator. The samples were resolved in 400 μl water and stored at −20°C.

### Analytical Procedures

#### MBTH Assay

De-*N*-acetylated osamines were detected and quantified by the MBTH assay (Plassard et al., 1982). The procedure was carried out in a 96 well plate (Sarstedt): 40 μl sample (containing up to 200 μg osamines/ml) were mixed with 40 μl 5 % KHSO_4_ (Sigma Aldrich). The samples were reduced by addition of 40 μl 5% NaNO_2_ (Sigma Aldrich) for 60 min at 50°C, whereby 40 μl 5% NaCl were used as negative control. After neutralization with 40 μl 12.5% NH_4_SO_2_NH_2_ (Sigma Aldrich) (ambient temperature, 30 rpm, 10 min), 40 μl 0.5% 3-Methyl-2-benzothiazolinon hydrazine hydrochloride hydrate (Sigma Aldrich) was added. The covered plate was incubated at 37°C for 30 min. Finally, 40 μl 0.5% FeCl_3_ was added and the absorption at λ = 650 nm was measured by the Tecan infinite M200Pro ELISA plate reader, while λ = 800 nm served as a reference wavelength. A calibration curve of a serial dilution of d-galactosamine (GalN) served as reference. To quantify the total amount of osamine or the degree of acetylation (DA), a hydrolysis step was performed prior the MBTH assay. Samples were totally hydrolyzed by 4 M HCl at 100°C for 4 h and subsequently dried overnight in a desiccator.

#### Acetate Assay

The DA was also estimated by the enzymatic acetate assay. Samples (220 μl) were totally hydrolyzed by addition of 100 μl 12 M HCl (100°C, 4 h). Then, 100 μl were used for the osamine detection by MBTH assay as described above and another 100 μl were used for the acetate assay. After neutralization by addition of 75 μl 7 M NaOH and 75 μl 2 M MOPS (pH 7.5), samples were subjected to the Acetate Colorimetric Assay (Sigma-Aldrich) according the manufacturer's protocol. The absorption at λ = 450 nm was measured by the Tecan infinite M200Pro ELISA plate reader, while λ = 700 nm served as a reference wavelength. The acetate content in the hydrolyzed samples was determined by a calibration curve of 0.25–1.5 mM acetate standard solution.

#### Monosaccharide Identification and Quantification by Gas Chromatography

Samples (100 μg) were hydrolyzed in 500 μl 8 M HCl for hexosamine analysis or 4 M TFA for hexose analysis. *Meso*-inositol (4 μg) was used as internal standard and 50 μg Gal, GalNAc, and GlcNAc as external standards. After hydrolysis, reduction with BH_4_Na and acetylation samples were analyzed by GC on a Perichrom PR2100 instrument with a flame ionization detector using a capillary column (30 m × 0.32 mm) filled with a DB-1 (SGE) as described previously (Fontaine et al., 2011).

### Enzymatic Degradation of GAG Fractions

#### Production of a Recombinant Endo-α-1,4-Galactosaminidase

For the enzymatic degradation of GAG, a poly-GalN hydrolase of *Pseudomonas sp*. (Tamura et al., 1988), here named GAGnase, was produced in *Escherichia coli*. Prior to the total synthesis of the gene, the DNA sequence was codon-optimized for expression in *E. coli*. In addition, a histidine tag was added to the carboxyl terminus to facilitate subsequent purification (Figure S2). The gene was cloned into the pET28a (+) expression vector and transformed into the *E. coli* BL21 Gold expression strain. A culture in the exponential growth phase was induced with 1 mM IPTG (final concentration), followed by production for 4 h at 30°C. The protein could be found in the culture supernatant (it contains a signal of bacterial secretion), the cytoplasm and in the inclusion bodies. The enzyme present in the supernatant was purified using ProBond™ Nickel-chelating agarose beads (ThermoFisher) (ratio 0.5 ml beads/50 ml supernatant) (Figure S2). The final preparation was kept in a 20 mM HEPES buffer pH 7.4, 137 mM NaCl and stored in aliquots at −20°C.

#### Enzymatic Degradation of Oligosaccharides

The enzymatic hydrolysis of GAG was carried out as previously described (Tamura et al., 1992). In brief, 5 mg/ml GAG/dGAG or 1 mg/ml GAG oligosaccharides were dissolved in 50 mM NaAc, pH 6.0. The reaction (100 μl scale) was started by addition of 2 μg/ml GAGnase and further incubated for 2 h at 37°C. The GAGnase was quickly heat-inactivated (100°C, 5 min) and the degradation efficiency was estimated by the sugar reducing end assay using the PABA as reagent (Lever, 1972).

### MALDI-TOF Mass Spectrometry

MALDI-TOF spectra were analyzed in reflectron positive mode using a 4800 TOF/TOF spectrometer (Applied Biosystems, Framingham, MA, USA) equipped with a pulsed nitrogen laser (337 nm and frequency of 200 Hz). An average of 5,000 shots per spot was used for MS data acquisition. Samples were prepared by mixing directly on the target 0.5 μl of oligosaccharide solution in water (10–50 pmol) with 0.5 μl of 2,5-dihydroxybenzoic acid matrix solution (10 mg/ml in CH_3_OH/H_2_O, 50:50, V/V). The samples were dried at room temperature.

### Isolation of Peripheral Blood Mononuclear Cells (PBMC)

Blood samples from healthy donors were obtained from Etablissement Français du Sang Saint-Louis (Paris, France) with written informed consent as per the guidelines provided by the Institutional Ethics Committee, Institut Pasteur (convention 12/EFS/023). Human blood sample from healthy patients was diluted 1:1 with PBS (Gibco). In a 50 ml falcon tube, 15 ml of lymphocytes separation medium (Eurobio) were slowly overlaid by 30 ml of the blood dilution. The cells were separated by centrifugation at ambient temperature (20 min, 1800 rpm). The upper phase (buffer and plasma) was discarded and the PBMCs (2–5 ml) were collected. The cells were washed by addition of 40 ml PBS and subsequent centrifugation at ambient temperature (10 min, 1,500 rpm). The wash step was repeated (30 ml PBS; 10 min, 1200 rpm). The cells were finally suspended in 10–20 ml RPMI 1640 + Glutamax-I (Gibco) and counted in a hemocytometer (c-Chip DHC-M01; Digital Bio). The cells were finally diluted to a concentration of 1 × 10^7^ cells/ml in RPMI and stored on ice.

### Detection of Interleukin Production in PBMCs by Enzyme-Linked Immunosorbent Assay (ELISA)

PMBCs were seeded in a final concentration of 5 × 10^5^ cells in 200 μl RPMI 1640 + Glutamax-I supplemented with 10% human serum in a U-shape 96 well plate. Native and de-*N*-acetylated GAGs were tested in a final concentration 0.5, 1, and 5 μg/ml (according to the MBTH assay). All de-*N*-acetylated and per-*N*-acetylated oligosaccharides have been tested in a final concentration of 1, 5, 10, and 25 μg/ml. Lipopolysaccharides (LPS) from *E. coli* (10 ng/ml; SIGMA) served as positive control. The cells were stimulated by GAGs, oligosaccharides or LPS for 24 h at 37°C, 5% CO_2_. The plates were centrifuged at ambient temperature (200 g, 3 min) and the supernatants were collected. Supernatants were stored at −20°C upon use. Experiments were carried out with five different batches of GAG and GAG-oligosaccharides with a minimum of four blood donors. The detection of IL-1Ra in the supernatants was carried out using the DuoSet ELISA Kits (R&D Systems) according to the manufacturers' protocol. The supernatants were tested in a final dilution of 1:33. The absorption at λ = 450 nm was measured by the Tecan infinite M200Pro ELISA plate reader, while λ = 570 nm served as a reference wavelength. In order to take variable donor-dependent cytokine levels into account, induction by GAG and GAG oligosaccharides were calculated as ratios to induction by LPS: Cytokine levels induced by 10 ng/ml LPS were set to 100% in each biological replicate and cytokine levels by GAG or GAG oligosaccharides were estimated accordingly.

### Detection of Cytotoxicity by Lactate Dehydrogenase (LDH) Assay

Putative cytotoxicity of GAGs and GAG oligosaccharide was estimated as LDH release from PBMCs. PMBCs were seeded in a final concentration of 5 × 10^5^ cells in 200 μl RPMI 1640 + Glutamax-I supplemented with 10% human serum in a U-shape 96 well plate. GAGs and oligosaccharides were tested in a final concentration 1.0 and 5.0 μg/ml (amount according to MBTH assay of hydrolyzed samples). Lipopolysaccharides (LPS) from *E. coli* (10 ng/ml; SIGMA) served as negative control. Totally lysed cells TritonX100 served as positive control and reference. All supernatants were 1:5 diluted in PBS + 1% BSA (positive control 1:20) and LDH assay was carried out by the LDH Cytotoxicity Detection Kit (Roche) according to the manufacturer's protocol. None of these oligosaccharides were toxic to PBMCs (Figure S3), showing that IL-1Ra secretion was not associated to cell apoptosis or necrosis.

### GAG and Oligo-GAG Application in DSS-Treated Mice

DSS (Dextran sulfate sodium, 2.5% wt/vol, 36,000–50,000 kDa; MP Biomedicals) was administered in drinking water *ad libitum* for 7 days. Fresh solution was replaced on day 3. Mice were injected intraperitoneally with 1 mg/kg of GAG samples for 7 consecutive days after DSS treatment. Weight loss, stool consistency, and fecal blood were recorded daily. Upon necropsy, tissues were collected for histology and cytokine analysis. Colonic sections were stained with hematoxylin/eosin. Colitis disease activity index was calculated daily for each mouse based on weight loss, occult blood, and stool consistency. A score of 1–4 was given for each parameter as in McNamee et al. (2011) To evaluate cytokine production, colons, opened longitudinally and washed in complete medium with antibiotics, were cultured at 37°C for 24 h in RPMI and 5% FBS. The supernatants were collected for ELISA and the remaining colonic explant tissue was homogenized and used for protein quantification (Quant-iT Protein Assay Kit, Life Technologies). The concentration of secreted cytokines in the supernatant was subsequently normalized to total tissue protein and expressed as picogram of cytokine per microgram of tissue. TNF-α, IL-1β, IL-17A, IL-17F, IL-10, and IL-1Ra were measured in culture supernatants of 24 h cultures using commercially available ELISAs (R&D systems) according to the protocols supplied by the manufacturer.

## Statistical Analysis

All experiments were performed with at least five different batches of GAG and oligosaccharides and a minimum of four blood donors. All statistics of *in vitro* experiments were carried out with a two-tailed unpaired Wilcoxon–Mann–Whitney Test by the free-of-charge software EDISON-WMW (Marx et al., 2016). Statistical analyses from *in vivo* experiments (**Figure 5**) were performed with one- or two-way ANOVA. Data, from one experiment using 10 mice/group, were expressed as mean ± SD and were analyzed in triplicate using GraphPad Prism Software. *P* values are indicated by asterisk as follows: ^*^p < 0.05; ^**^p < 0.01; ^***^p < 0.001.

## Ethical Statement

Murine experiments were performed according to the Italian Approved Animal Welfare Authorization 360/2015-PR and Legislative degree 26/2014 regarding the animal license obtained by the Italian Ministry of Health lasting for five years(2015-2020).

## Results

### Soluble Long α-1,4-Galactosamine Oligomers (DP > 20) From GAG Induce IL-1Ra on PBMCs

**Figure 1:**
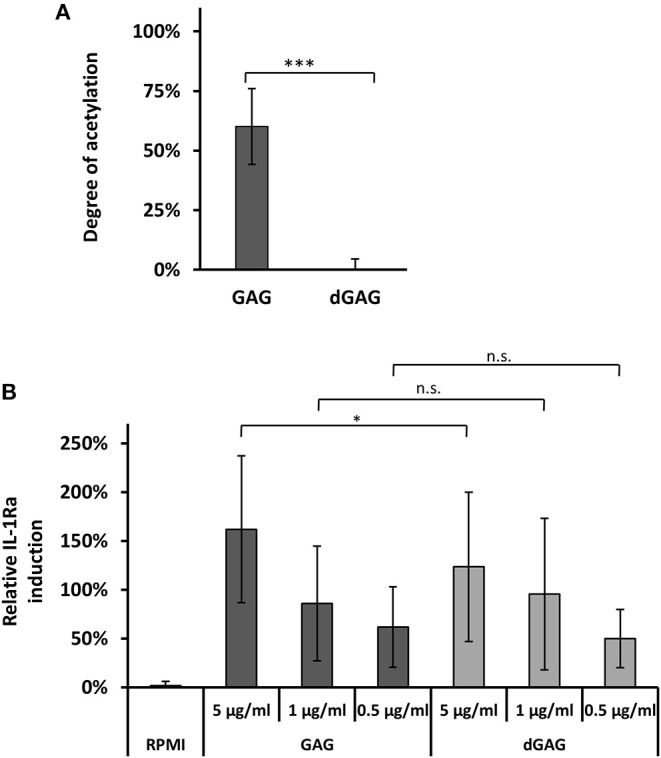
De-*N*-acetylation of GAG. Degree of acetylation (DA) of GAGs **(A)**. DA was determined by an acetate assay (GAG, native polymer; dGAG, de-N-acetylated) **(B)**. *In vitro* IL-1Ra secretion in PBMC supernatants in presence of GAG. LPS (10 ng/ml) served as external standard and were set to 100% in each biological replicate. Statistical analysis by WMW test in comparison to GAG: ns, non significative; *p < 0.05; ***p < 0.001.

**Figure 2:**
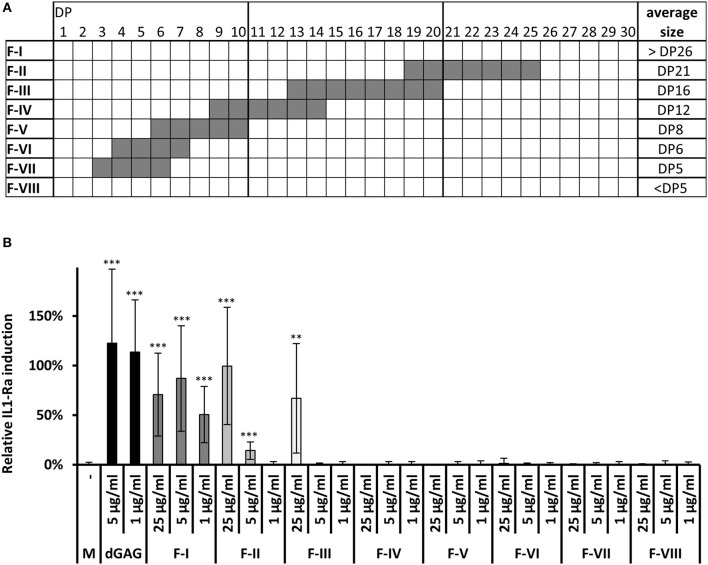
Chemical analysis and IL-1Ra inducing activity of oligosaccharides generated by acid hydrolysis of dGAG. **(A)** Summerized MALDI-TOF MS analysis of fractions I-VIII. (DP, degree of polymerization; for detailed MS spectra see Figure S2). **(B)** IL-1Ra production by dGAG oligosaccharides. dGAG oligosaccharides fractions I-VIII were tested at 25, 5, and 1 μg/ml. dGAG (5 and 1 μg/ml) served as positive control. LPS (10 ng/ml) served as external standard and were set to 100% in each biological replicate. Wilcoxon-Mann-Whitney Test: **p < 0.01; ***p < 0.001 (compared to control, M).

Whole GAG fractions, isolated from Brian culture supernatant of *A. fumigatus*, were composed of galactose, galactosamine, and *N*-acetylgalactosamine residues. A degree of acetylation (DA) of galactosamine was determined to be 66 ± 7%, which is in agreement with previous observations (Lee et al., 2016). To further investigate the impact of the DA on IL-1Ra induction, the GAG polymer was de-*N*-acetylated by a harsh alkali treatment (4 h, 100°C, 10 M NaOH) (No and Meyers, 1989) to produce a fully de-*N*-acetylated GAG (dGAG). GC analysis of monosaccharide revealed that the galactose content in native GAG (%Gal 8.3 ± 0.2%) was slightly reduced in dGAG (2.3 ± 2.0%) (Figure S1). Both native GAG and dGAG induced IL-1Ra in a concentration dependent manner to similar extent (Figure 1). To produce and isolate soluble oligosaccharides of different size, dGAG was hydrolyzed in 2 M HCl for 1.5, 3, or 4.5 h at 100°C. After the removal of acid by under vacuum, water-soluble oligomers were recovered by centrifugation and separated by size exclusion chromatography (SEC) on a Superdex 30 column (Figure S1). While 1.5 h hydrolysis mainly resulted in the production of larger oligosaccharides, 3 and 4.5 h hydrolysis times induced the release of small-size oligosaccharides. Resulting of pooled oligomer fractions from the three hydrolysates (fractions I-VIII) were analyzed by MALDI-MS and GC (Figures S1, S4 and Figure 2A). Fractions V till VII contained oligosaccharides till DP10 with the presence of GalN and Gal residues and traces of glucose. Fractions II to IV were characterized by higher MW oligosaccharides till DP26 containing only GalN residues (Figure 2A and Figure S4). Fraction I, composed also exclusively of GalN residues did not give any ion peak by MALDI-TOF showing that this fraction contained larger oligosaccharides (dp > 26). All these 8 fractions were tested as putative IL-1Ra inducers (Figure 2B). Oligomers of dp < 13 (Fractions IV to VIII) did not induce IL-1Ra expression at the tested concentrations. A moderate activation was observed for fraction III (13 < dp < 20) at the concentration of 25 μg/ml, but not at lower concentration (5 and 1 μg/ml). Fractions I and II stimulated IL-1Ra production at 1 and 5 μg/ml, showing that galactose-free, de-*N*-acetylated oligosaccharides are potential inducers. Fraction I induced IL-1Ra production at a similar extend as the GAG native polymer showing that larger were the soluble GalN oligosaccharides better was the production of IL-1Ra.

**Figure 3:**
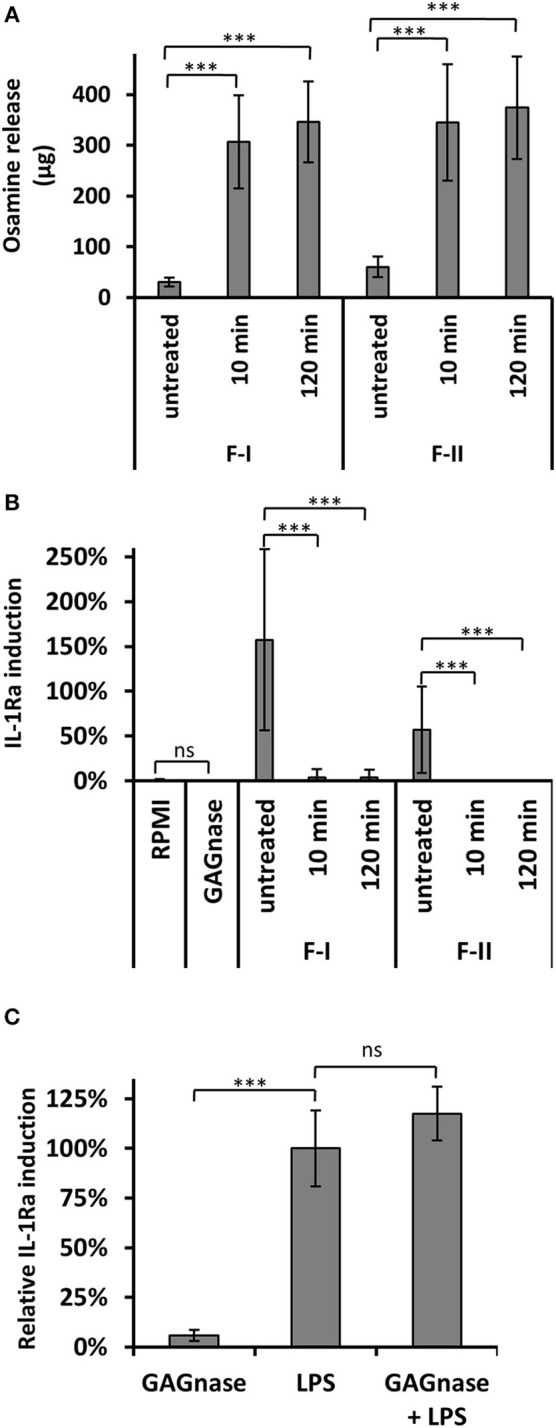
IL-1Ra stimulation by GAG oligomers is abolished by GAGnase. **(A)** Fraction F-I and F-II are degraded by GAGnase. Fractions I and II (500 μg/ml) were enzymatically hydrolyzed by GAGnase (2 μg/ml) for 10 or 120 min or were kept in acetate buffer for 120 min (untreated). The degradation was followed by PABA assay to quantify sugar reducing end. **(B)** Induction of IL1-Ra expression in PBMCs by GAGnase treated fractions. **(C)** IL-1Ra induction of PBMCs in presence of GAGnase (20 ng/μl). GAGnase neither activate nor inhibit induction. LPS (10 ng/ml) served as reference control and were set to 100% in each biological replicate. bars indicate the SD, WMW Test: ***p < 0.001; ns, not significant (compared to control samples).

Fractions I and II were subjected to an enzymatic degradation with a recombinant endo-α-1,4-polygalactosaminidase (GAGnase) from *Pseudomonas sp*. (see material and methods for the production of the recombinant enzyme and Figure S2), which specially hydrolyzed α-1,4-linked GalN polymers to release GalN oligomers of DP 2 and 3, but does not use poly-*N*-acetyl-galactosamine or other polysaccharides as substrate (Tamura et al., 1988, 1992). Fractions I and II were degraded by the recombinant GAGnase as determined by the release of reducing ends (Figure 3A). Moreover, the hydrolyzed fractions lost their ability to induce IL-1Ra secretion in PBMCs, showing that only long α-1,4-GalN oligomers are inducers (Figure 3B). The GAGnase protein neither activates nor inhibits IL-1Ra expression at the desired concentration (Figure 3C).

### Deacetylation of Galactosamine Oligomers Is Essential to Induce the Production of IL-1Ra

**Figure 4:**
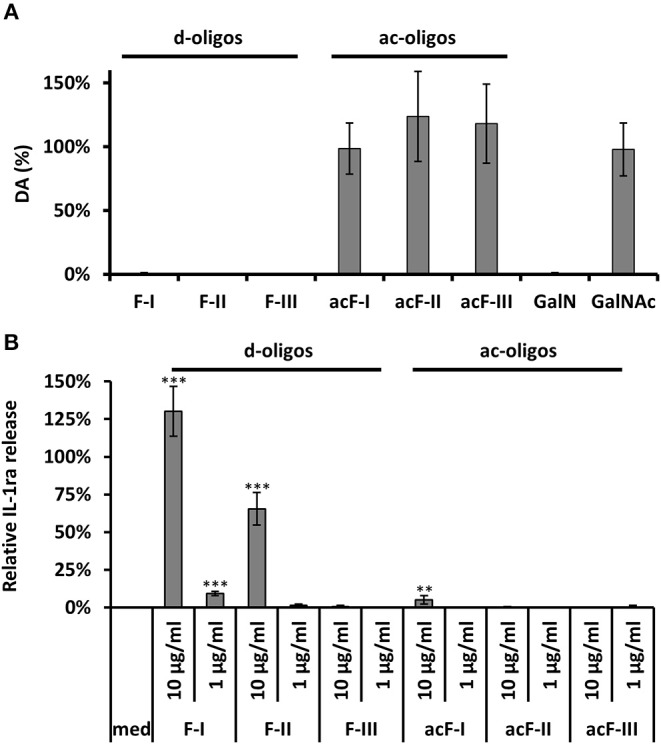
*N*-acetylation of de-*N*-acetylated GAG oligosaccharides and its impact on the IL-1Ra production. **(A)** Determination of the degree of acetylation (DA) by acetate assay of de-*N*-acetylated and acetylated fractions I, II and III. GalN and GalNAc served as positive and negative controls. **(B)** *In vitro* IL-1Ra production by de-*N*-acetylated and acetylated GAG fractions I, II, and III (10 and 1 μg/ml). LPS (10 ng/ml) served as positive control. WMW Test: **p < 0.01, ***p < 0.001.

To test the impact of acetylation of GAG-oligosaccharides on PBMC stimulation, GalN oligomers (fraction I to III) were *N*-acetylated by acetic anhydride. After the acetylation procedure, the per-*N*-acetylation and the absence of degradation by GAGnase were confirmed by acetate assays (Figure 4A) and MALDI-TOF-MS (Figure S5). A water-insoluble material was observed in fractions I and II and but not in fraction III, showing a decrease of solubility for the larger *N*-acetylated oligomers. Most importantly, per-*N*-acetylated fractions I and II have lost their capability to induce IL-1Ra (Figure 4B). A slight induction of IL-1Ra was seen with the insoluble re-*N*-acetylated fraction I which may be due to the presence of insoluble particles which can induce unspecifically cytokine production (Becker et al., 2016). Taking together, our data provided evidence that soluble large GalN oligomers specifically triggered the IL-1Ra induction *in vitro*.

### Soluble Long De-*N*-Acetylated GAG Oligomers Ameliorate Colitis in Dextran Sulfate Sodium (DSS)-Treated Mice

**Figure 5:**
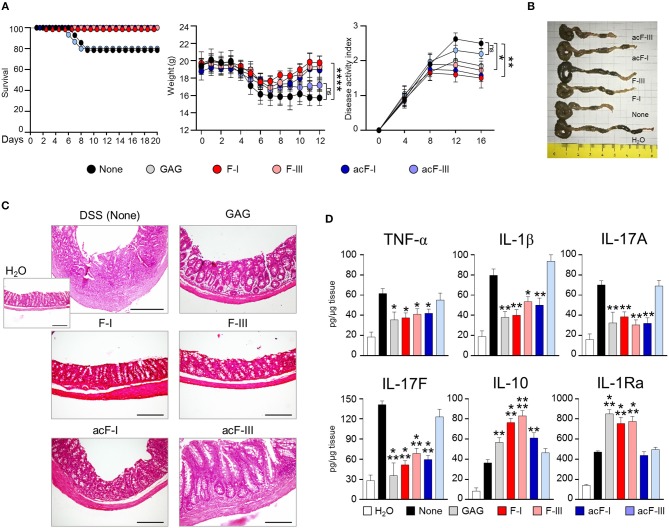
Reduced severity of DSS colitis in C57BL/6 mice treated with modified GAG molecules. DSS or water vehicle was administered *ad libitum* in drinking water for 7 days. Native GAG polymer and GAG oligomers at the dose of 1 mg/kg were given intraperitoneally for 7 consecutive days after DSS treatment. **(A)** Survival, weight (grams) and clinical disease activity index. **(B)** Macroscopic image of colons. **(C)** Histological assessment of colitis severity (20X magnification, bar = 200 μm). **(D)** Levels of colonic cytokines. Data are expressed as mean ± SD. *P < 0.05, **P < 0.01, ***P < 0.001, ****p < 0.0001 GAGs-treated vs. untreated (None) C57BL/6 mice (*n* = 10 mice/group from one experiment). ns, not significant.

To test the *in vivo* immunosuppressive potential of GAG derivates, oligomers were tested in the experimental dextran sulfate sodium (DSS)-induced colitis model in C57BL/6 mice (Gresnigt et al., 2014). DSS treated mice lost over 25% body weight, showed a reduced survival, an elevated DAI (disease activity index) and colonic injury observed by macroscopic imaging. Fractions I (>dp26) and III (13 < dp < 20) as well as their *N-*acetylated counterparts have been injected to DSS-treated mice (Figure 5). Intraperitoneal injections of both versions of F-I led to ameliorating the clinical signs of colitis by increasing animal survival, reducing weight loss and DAI (Figure 5A), colon size and histological alterations showing the amelioration of colonic injury (Figure 5). Cytokine profile, such as a decrease of IL-17A and IL-17F and increase of IL-1Ra, showed that the deacetylated version (F-I) rescued DSS treated mice in a similar way as the whole GAG polymer. In addition, F-I also promoted IL-10, a cytokine known to be protective in colitis because IL-10-deficient mice spontaneously develop enterocolitis and colon cancer (Wang et al., 2015). The per-*N*-acetylated fraction I (acF-I) did not induce IL-1Ra, suggesting that *N*-acetylated F-I has a beneficial effect through an IL-1Ra independent pathway. Acetylated and de-*N*-acetylated smaller oligosaccharides (acF-III and F-III) showed opposite effects *in vivo*. IP injection of F-III rescued the mice from death and the weight loss, ameliorated DAI and strongly reduced inflammatory lesions (Figure 5). At the cytokine level, F-III induced a similar pattern of cytokine secretion as F-I with a decrease of pro-inflammatory cytokine and increase of anti-inflammatory interleukins such as IL-10 and IL-1Ra (Figure 5D). Since F-III was a poor IL-1Ra inducer *in vitro* (Figure 2B), our data suggested that the size of GalN oligomers was less crucial *in vivo* than *in vitro*. In contrast, the administration of the acetylated counterpart, acF-III, did not rescue the inflammation where all colitis characteristics (death, loss of weight, DAI, colon analysis, cytokine pattern) were similar to those of DSS control mice (Figure 5). Even worse, acF-III administration increase pro-inflammatory cytokines such as TNFα, IL-1β, and IL-17 (Figure 5).

Taken together, data from Figure 5, two colitis rescue profiles were observed with GAG oligomers. De-*N*-acetylated oligomers (average dp of 16 or larger) rescued DSS-treated mice with a significant reduction of inflammatory cells infiltration, muscle thickening and amelioration of colonic structure. This effect is concomitant to the induction of IL-1Ra secretion as also observed *in vitro*. In contrast, insoluble acetylated oligomers of dp > 26 rescued DSS-treated mice in a IL-1Ra-independent mode of action. In summary, water-soluble poly-galactosamine oligosaccharides are promising candidates for alternative treatments of IL-1Ra-dependent diseases.

## Discussion

IL-1-mediated inflammation has been established in a broad spectrum of diseases, ranging from rare autoinflammatory diseases to common conditions such as gout and rheumatoid arthritis, type 2 diabetes, atherosclerosis, and acute myocardial infarction (Cavalli and Dinarello, 2018). The search for potent IL-1 blockers to treat major inflammatory diseases has turned into a very active field, which addresses several therapeutic approaches (Finckh and Gabay, 2008; Gabay et al., 2010). Among them, prevention of binding of active IL-1 on its cell surface receptors by use of the recombinant form of IL-1Ra (Anakinra) offers an ideal option as it has been used in several diseases (Dinarello et al., 2012). Due to its anti-inflammatory potential, GAG produced by *A. fumigatus* opens the way to novel innovative strategies to control IL-1 signaling and to design new anti-inflammatory drugs (Gresnigt et al., 2014). Here, to investigate the minimum oligosaccharide sequences derived from GAG able to induce anti-inflammatory response, partial chemical hydrolysis allowed isolating soluble active oligomers. From our data, galactose is not required for the bio-activity of GAG, in contrast, de-*N*-acetylated GalN residues were essential to induce the secretion of IL-1Ra both *in vitro* and *in vivo*. The size of oligomers plays a critical function where a minimum size of 13 (and better >19) α-1,4-GalN residues were required to be active *in vitro*. *In vivo* experiments have confirmed the *in vitro* data and are able to rescue inflammatory damage in a colitis model. Interestingly, the poor *in vitro* inducer F-III has a similar *in vivo* beneficial effect than larger oligomers, suggesting that the dose/size effect of GalN oligomers is different *in vitro* and *in vivo*. The chemical synthesis of defined GalN oligomers and *in vivo* kinetic studies will be helpful to investigate the optimal size and the delivery of such molecule *in vivo*.

The question of the chemical specificity of the IL-1Ra inducers by cell wall polysaccharides is now fundamental. Cell wall chitin and β-glucans are also IL-1Ra inducers (Poutsiaka et al., 1993; Smeekens et al., 2015; Becker et al., 2016). β-glucan structures remain essential to the IL-1Ra induction since soluble forms are not inducers (Poutsiaka et al., 1993). This result is in agreement with our data indicating the differential immunological effect of soluble and insoluble polysaccharide species. Moreover, β-glucans isolated from yeast or the hyphal form of *Candida albicans* did not induce similar effects (Smeekens et al., 2015) suggesting that small changes in the chemical composition of the polysaccharides can modify the immune response. Pure chitin isolated from *A. fumigatus* cell wall induces IL-1Ra (Becker et al., 2016). Chitin is composed of *N*-acetylglucosamine residues. However, in contrast to GAG, it is the *N*-acetylated form of glucosamine residues that is required for the IL-1Ra induction (Becker et al., 2016 and data not shown). The seemingly contradictory literature on the immunostimulatory properties of chitin are likely due to many factors including differences in sources of material, purity, readouts for inflammatory responses and the size of particles. Large chitin particles strongly induce pro-inflammatory response whereas small ones are not immunogenic (Alvarez, 2014). The degree of acetylation of cationic polysaccharides such as chitosan is critical for the pro-inflammatory response, showing that the de-*N*-acetylation of osamines not only alter their physicochemical properties but also their immunological activities (Bueter et al., 2011). The respective anti-inflammatory and pro-inflammatory properties of N-acetylated F-III and de-N-acetylated F-III are also an illustration (Figure 5). Chitin plays a dual immune response depending of the purity: (1) pure chitin induces an anti-inflammatory response in presence of serum and (2) in presence of other PAMPs (LPS, muramyl dipeptide, but not β-glucans), chitin has a synergistic effect on the induction of pro-inflammatory cytokines in an independent pathway (Becker et al., 2016), showing that the purity and homogeneity of PAMP sample are extremely important to investigate immune response.

β-Glucans induces both inflammatory and anti-inflammatory response that are triggered through independent pathways. Two main PPRs, the C-type lectin Dectin-1 and the complement receptor CR3 are known to recognize β-glucans (Brown and Gordon, 2001; van Bruggen et al., 2009). In *A.fumigatus* the pathway linking GAG to IL-1Ra production is unknown. IL-1Ra induction by *C. albicans* β-glucans is dependent on a Akt-PI3K dependent PRR (Smeekens et al., 2015). Our data showed that insoluble and soluble GAG oligomers shared an anti-inflammatory response with two independent pathways. Insoluble oligomers may restore a protective effect *in vivo* on colitis through an IL-1Ra independent pathway. In contrast, soluble oligomers induce a specific induction of IL-1Ra where the presence of de-*N*-acetylated galactosamine residues is critical. Although the signaling pathway remains unknown, it is independent on particle size or insolubility of polymer as described for chitin and β-glucans. Our study showed that soluble oligomers composed of an average of 16 α-1,4-linked GalN residues display a specific IL-1Ra induction, suggesting a specific immune pathway. Interestingly, pectin macromolecules have gained significant attention partly because of their immunosuppressive properties (Popov and Ovodov, 2013). Pectin induced *in vitro* a dose-dependent inhibition of IL-1β secretion correlated to an increase secretion of anti-inflammatory cytokines IL-10 and IL-1Ra by human PBMCs (Salman et al., 2008). The anti-inflammatory properties are dependent on a high content of α-1,4-linked galacturonic acid in the main polysaccharidic chain (Popov and Ovodov, 2013). The structural comparison with GAG from *A. fumigatus* suggests a structure-function relationship where linear α-1,4-linked galactose derived monosaccharides may be important for the anti-inflammatory properties of the polymer. However, our data showed that GAG oligomers induce IL-1Ra in PBMCs to a 20–100 fold extend *in vivo* and *in vitro* as compared to pectin (Salman et al., 2008). α-1,4-GalN oligomers are highly water-soluble alternatives to ease medically dose-response-adjustments and to overcome application issues frequently observed for insoluble chitin/chitosan polymers (Vo and Kim, 2014). Finally, our data suggest that the chemical synthesis of α-1,4-GalN oligomers depict new tools to investigate the specific immune pathway of GAG and provide a novel source of anti-inflammatory glycodrugs for treatment of IL-1-driven diseases.

## Data Availability Statement

All datasets generated for this study are included in the article/Supplementary Material.

## Ethics Statement

The animal study was reviewed and approved by the Italian Animal Welfare Authorization 360/2015-PR and Legislative degree 26/2014.

## Author Contributions

MG, CH, RS, LR, J-PL, and TF conceived and designed the experiments. MG, CH, IN'G, GR, VO, SM, BC, JG, and TF performed the experiments. MG, CH, BC, RS, LR, JP-L, and TF analyzed the data. MG, CH, GR, VO, SM, BC, JG, RS, LR, J-PL, and TF contributed reagents, materials and analysis tools. MG, TF, and J-PL wrote the paper.

### Conflict of Interest

The authors declare that the research was conducted in the absence of any commercial or financial relationships that could be construed as a potential conflict of interest.
