# Supplementary material for: Definition of the Anti-inflammatory Oligosaccharides Derived From the Galactosaminogalactan (GAG) From Aspergillus fumigatus
Source: Front Cell Infect Microbiol. 2019 Nov 6;9:365. doi: 10.3389/fcimb.2019.00365 (PMC6851199; doi:10.3389/fcimb.2019.00365)
Supplement: Supplementary file 1 [file Data_Sheet_1.PDF]

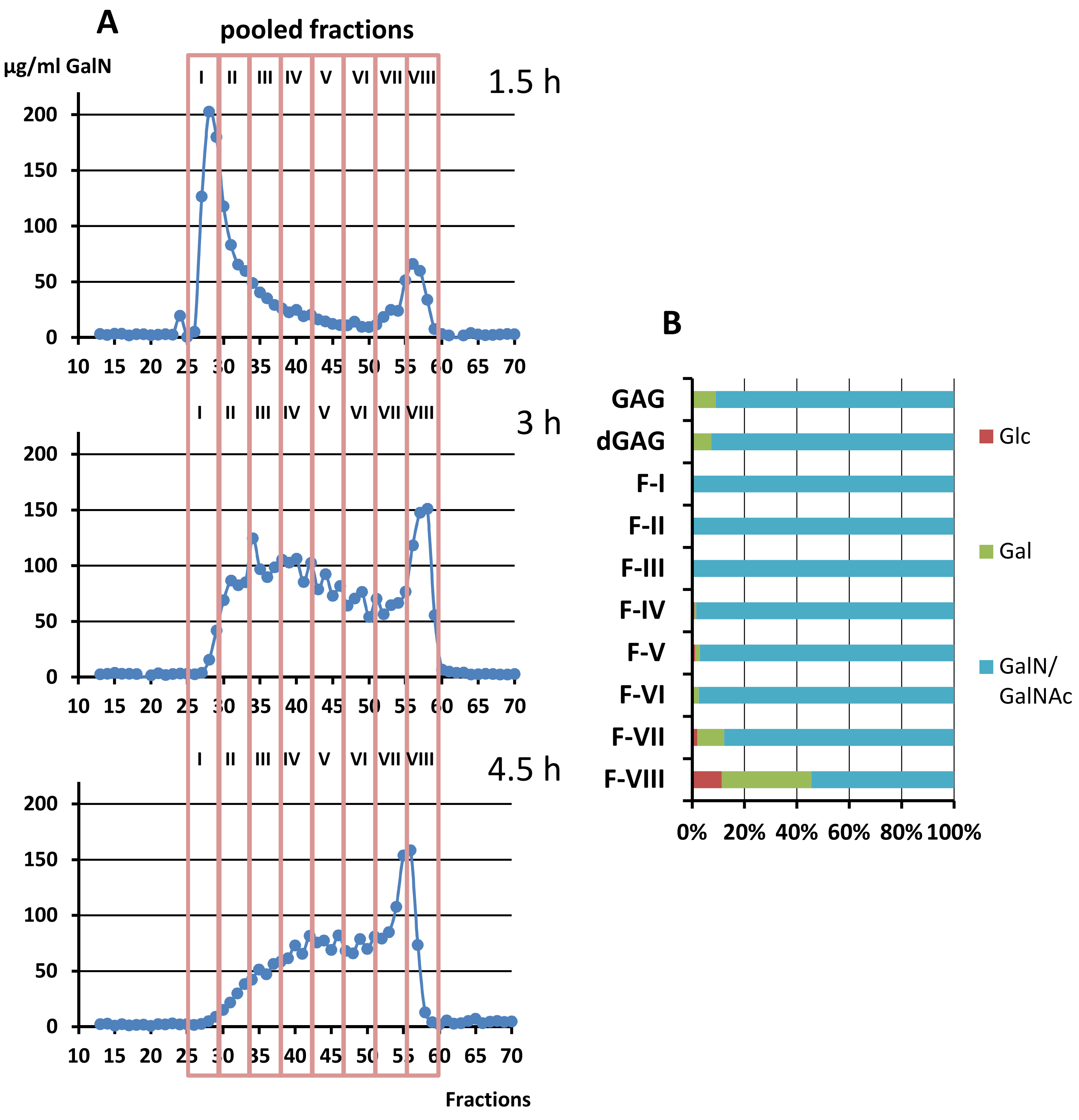

Figure S1. Separation and composition of dGAG hydrolysates. dGAG was hydrolyzed by 2 M HCl for 1.5, 3 or 4.5 h at 100°C. **A**, Hydrolysates were subjected to size-exclusion chromatography (SEC) on a Superdex 30 column. Retention was assessed by MBTH assay. Pooled fractions I-VIII were indicated as red boxes. **B**, GC monosaccharide analysis of fractions I-VIII and dGAG. (Glc, D-Glucose; Gal, D-Galactose; GalN/GalNAc, D-Galactosamine/N-Acetyl-D-Galactosamine).

A

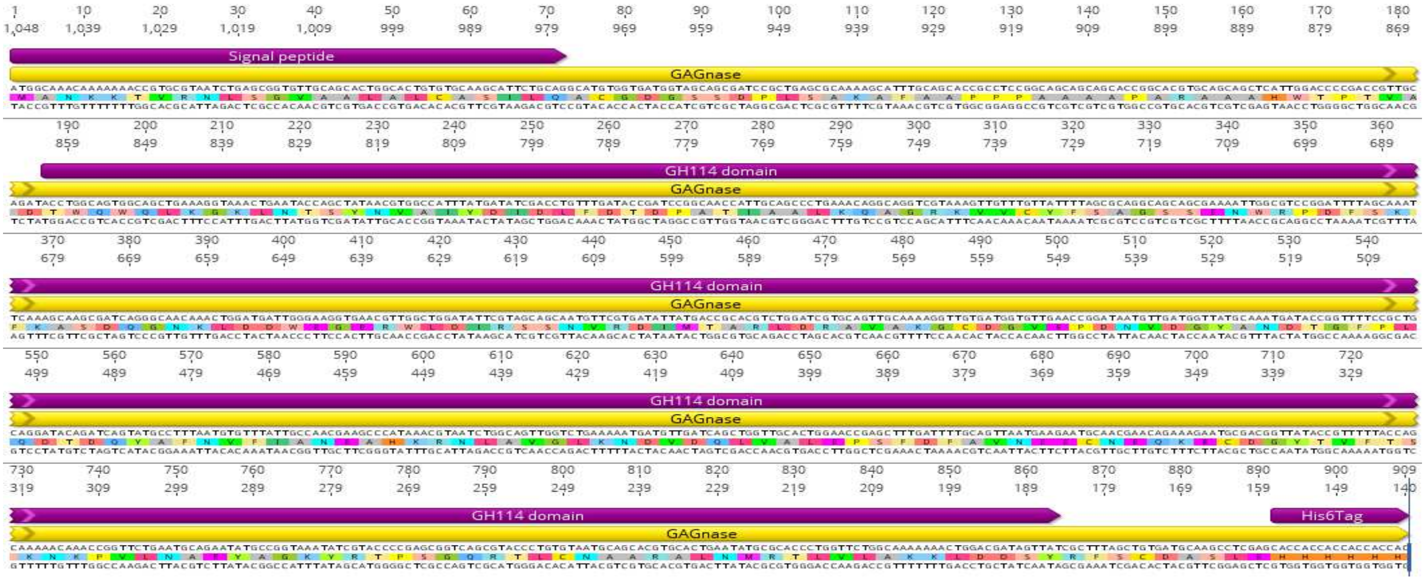

B

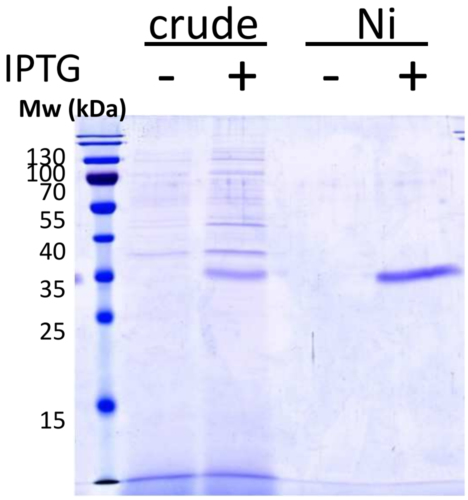

expected size: 33.2 kDa

Figure S2. Purification of *Pseudomonas* endo-α-1,4-galactosaminidase (GAGnase) from *E. coli* BL21 Gold transformed with GAGnase_pET28a(+). **A.** Nucleotide sequence of the *E. coli* codon-optimized *Pseudomonas* GAGnase gene including its His-Tag. **B.** SDS-PAGE of total protein extracts and purified GAGnase from *E. coli* with (+) and without (-) induction by 1 mM IPTG. Purification from culture broth supernatant revealed a high purity of the GAGnase fraction . Crude; crude culture broth supernatant; Ni, fraction after purification with Ni-NTA beads.

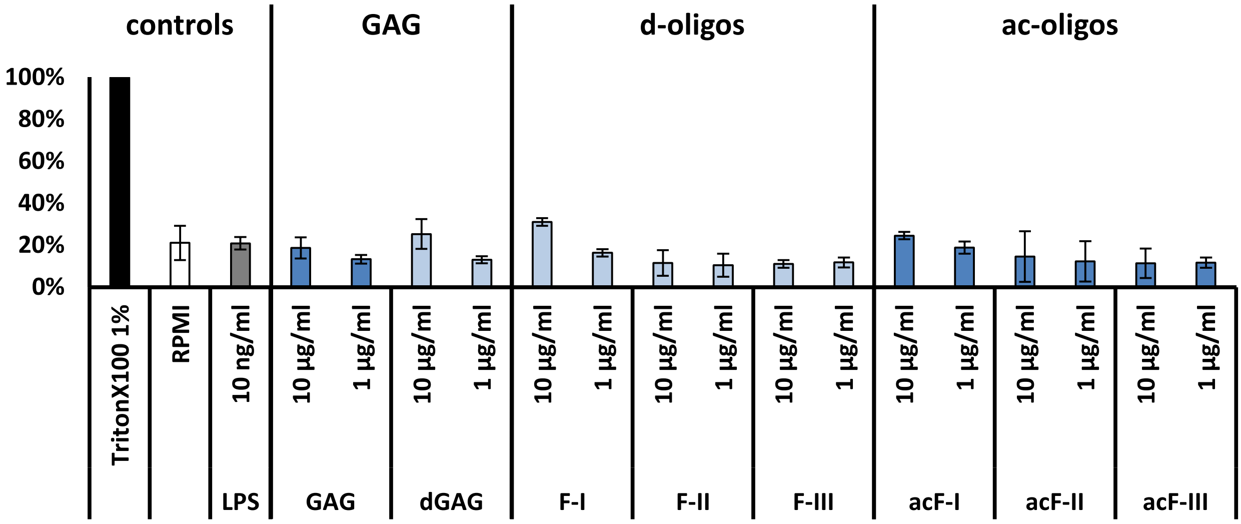

Figure S3. Cytotoxicity of oligosaccharides. Cytotoxicity was estimated as LDH release from PBMCs in presence of GAGs and GAG oligosaccharide fractions I, II and III. All values are given as ratios to total lysis control (1% TritonX100). Non-induced (RPMI) and LPS-stimulated (10 ng/ml) cells served as negative control.

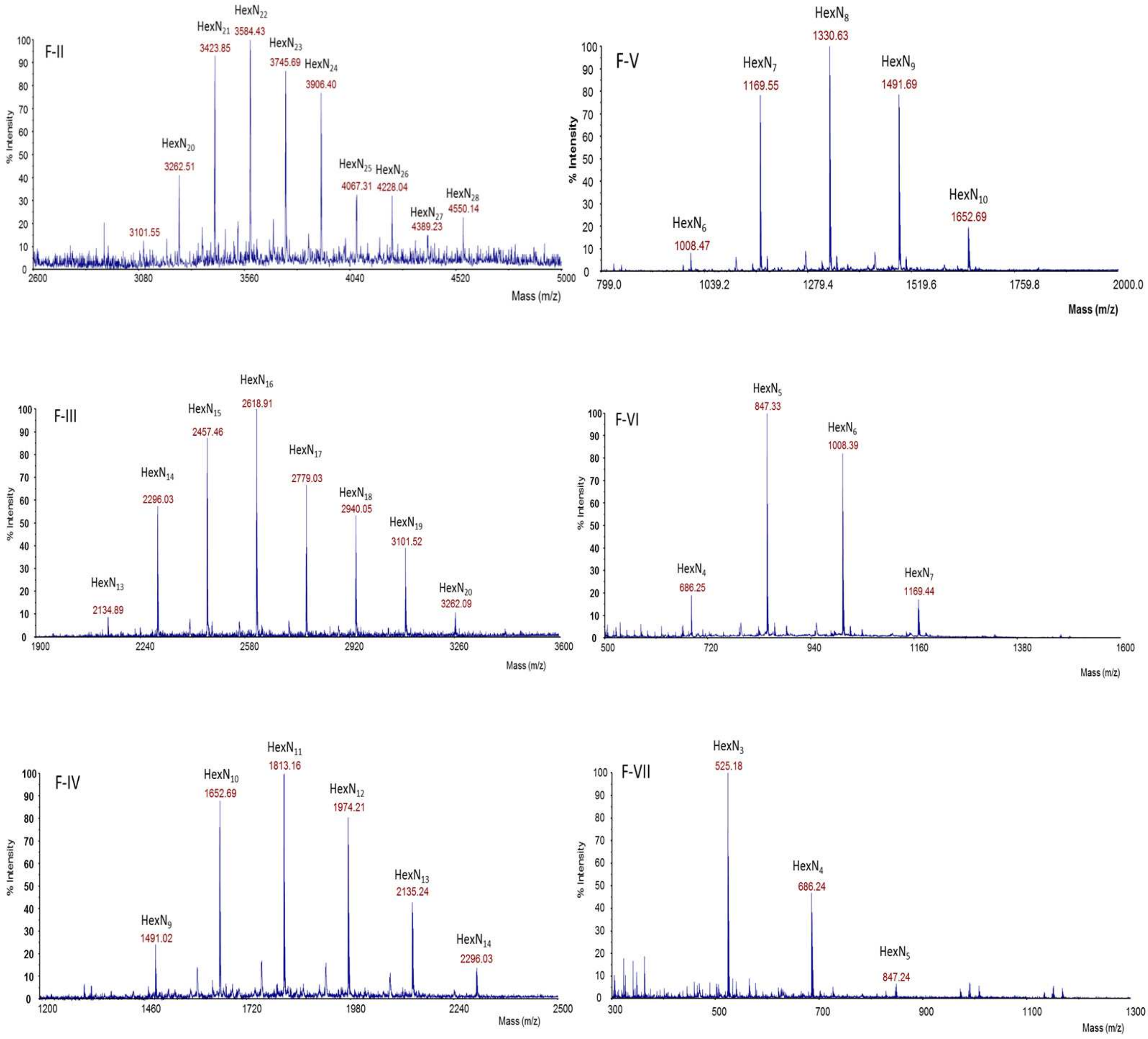

Figure S4. Positive MALDI-TOF spectra of fractions II-VII from hydrolysed dGAG (2M HCl, 100°C) and subsequent SEC. Masses (m/z) correspond to pseudomolecular ions $[M+Na]^+$-H_2O. The degree of polymerization of hexosamine ($HexN_n$) oligomer peaks is indicated. Note that fraction I (not shown) did not result in a defined MS spectra regardless the ionization method or spotting technique and fraction VIII was contaminated with salts impeding MALDI-MS analysis.

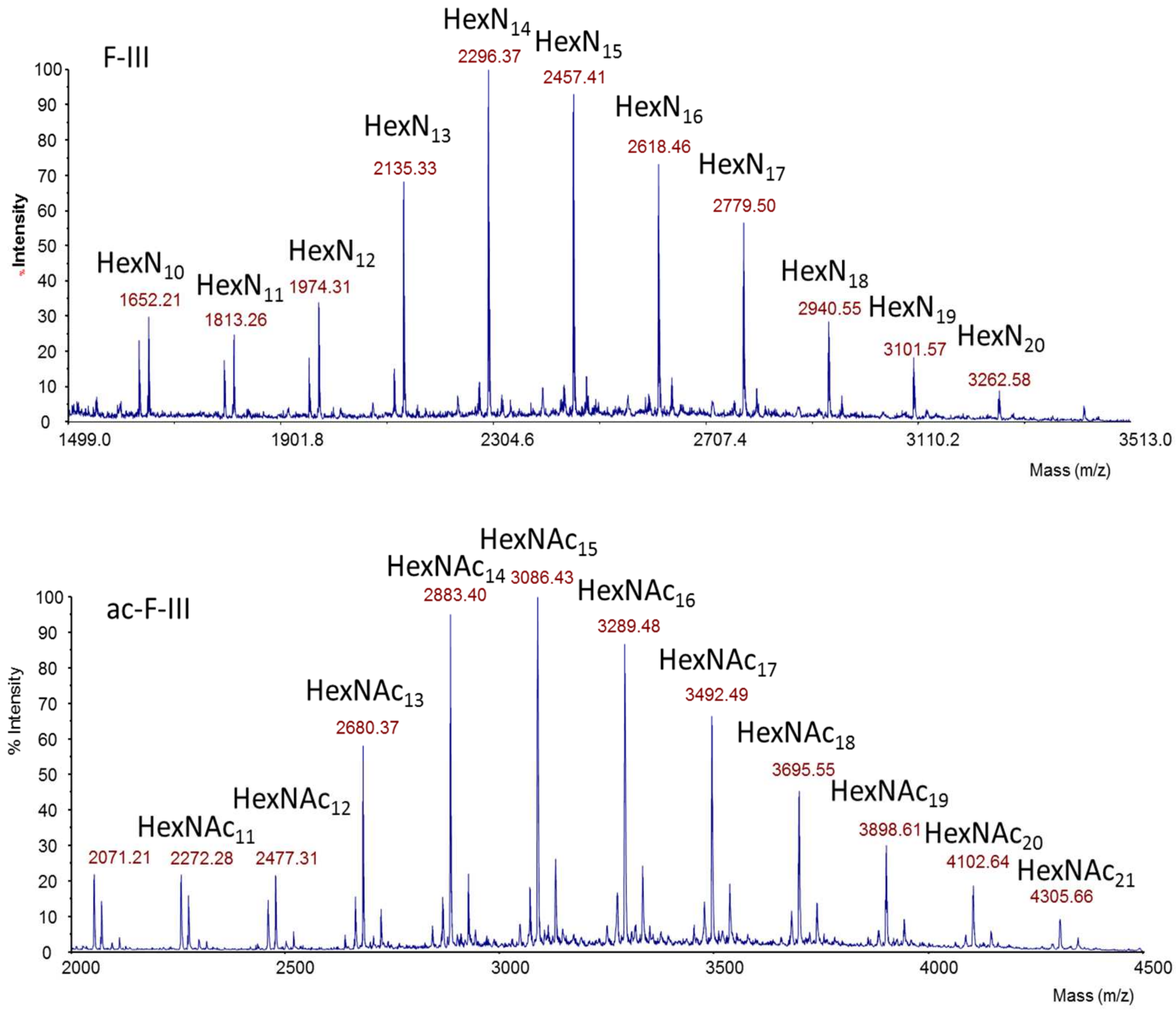

Figure S5. Positive MALDI-TOF mass spectra of oligomers fraction III before and after *N*-acetylation. MS spectra of oligosaccharides of fraction III prior (F-III) and after complete *N*-acetylation (ac-F-III). Masses (m/z) correspond to pseudomolecular ions $[M+Na]^+$. As shown in Fig. S3, de-*N*-acetylated oligmers have lost a water molecule ($-H_2O$) during MS analysis. The indicated degree of polymerization of hexosamine ($HexN_n$ or $HexNAc_n$) oligomer peaks show that per-*N*-acetylation did not cause oligomer degradation.
